# Loss of ARID1A expression is associated with worse survival and reduced tumor infiltrating lymphocytes in advanced clear cell renal cell carcinoma

**DOI:** 10.1007/s12094-025-04166-8

**Published:** 2026-01-03

**Authors:** Mohamed Ashraf Mansour, Reshmi Patel, Faiz Mumtaz, Ekaterini Boleti, Axel Bex, Maxine Gia Binh Tran, Soha El-Sheikh

**Affiliations:** 1 Department of Cellular Pathology, Royal Free London Foundation Trust, London, UK Pond Street, NW3 2QG; 2 School of Clinical Medicine, University of Cambridge, Cambridge, UK; 3 Specialist Centre for Kidney Cancer, Royal Free London Hospital, London, UK; 4 UCL Division of Surgery and Interventional Science, Rowland Street, London, UK; 5 Department of Urology, The Netherlands Cancer Institute, Amsterdam, The Netherlands; 6 Research Department of Pathology, University College London (UCL) Cancer Institute, London, UK

**Keywords:** ARID1A, Renal cell carcinoma, Immunohistochemistry, Tumor infiltrating lymphocytes, Immunotherapy

## Abstract

**Background:**

Patients with metastatic clear cell renal cell carcinoma (ccRCC) are often treated with immunotherapy (ICI), with no definitive biomarkers of response. *ARID1A* is mutated among ICI responders in several cancer types, including ccRCC. Immunohistochemistry (IHC) is a widely available method of detecting ARID1A protein levels. Assessment of tumor infiltrating immune cells (TILs) also has been linked to ICI response in some studies.

**Patients and methods:**

We explored the expression of ARID1A protein using IHC (manual and QuPath) in a cohort of 29 patients with metastatic ccRCC who had undergone cytoreductive nephrectomy (CRN). We correlated ARID1A expression with clinicopathological data, survival, and TILs. We corroborated our IHC results in 488 cases from the TCGA-KIRC dataset, and utilized immune deconvolution platforms to define changes in TILs in relation to ARID1A in TCGA-KIRC and an ICI-therapy validation cohort.

**Results:**

Low ARID1A protein expression is associated with large tumor size, lymphovascular invasion, high stage, low TILS, and worse overall survival. Low ARID1A mRNA in TCGA-KIRC similarly had significantly worse survival and were immune cold histologically and in ESTIMATE immune score. xCell showed significant enrichment of Th1, Th2, myeloid dendritic cells, macrophages, and T NK cells in low ARID1A mRNA ccRCC with elevation of Tregs in tumors that have high ARID1A.

**Conclusion:**

Low ARID1A expression (protein and mRNA) is a marker of poor prognosis in ccRCC, and is associated with shorter survival and reduced TILs, but immune cells linked to ICI response are increased offering an immune advantage to ARID1A^Low^ patients who receive ICI therapy. ARID1A IHC provides comparable results to bulk mRNA analysis, suggesting that it can be reliably used to explore ARID1A as a potential ICI biomarker in ccRCC.

**Supplementary Information:**

The online version contains supplementary material available at 10.1007/s12094-025-04166-8.

## Background

Over 430,000 new cases of renal cell carcinoma (RCC) were reported worldwide in 2022, representing 2.2% of all cancer diagnoses, with 156,000 deaths per annum [1]. Around 20–25% of patients diagnosed with RCC annually present with metastatic disease [2], and are directed to systemic targeted therapy in the form of tyrosine kinase inhibitors (TKI) and/or immune check point inhibitors (ICI), with or without cytoreductive nephrectomy (CRN) to resect/debulk the primary tumor [3]. Despite the huge improvements in patient outcomes achieved by ICI, the efficacy of immunotherapy varies among ccRCC patients. Predictive biomarkers approved in other tumor types such as PD-L1 staining and a high tumor mutational burden were shown not to be clinically relevant in RCC [4].

There is mounting evidence that tumors with alterations in the Switch/Sucrose Non-Fermentable (SWI–SNF) chromatin remodeler complex genes may predispose to improved outcomes with ICI [5]. *PBRM1*, a member of the SWI–SNF complex, is the second most frequently mutated gene in ccRCC [6]. Initial reports suggested that *PBRM1* mutations in ccRCC may offer clinical benefit to ICI [7], but subsequent studies failed to confirm *PBRM1* mutations as a positive predictive marker for response to ICI [8].

*ARID1A,* another member of the SWI–SNF complex, was shown to act as a tumor suppressor gene in RCC which, when silenced using siRNAs, reduces apoptosis, increases cell proliferation, and increases invasiveness [9]. In the IMmotion151 (a clinical trial that included 823 patients with advanced RCC), mutations in *ARID1A* were significantly enriched among ICI responders with better progression-free survival, compared to those treated with TKIs [10]. This finding is not unique to RCC. In pan-cancer studies, ARID1A had the highest mutation rate across all components in the SWI/SNF complex [11], and out of 1660 patients who received ICI (including 151 RCC patients), ARID family members (including *ARID1A*) were considered as potential novel biomarkers for ICI [12].

The frequency of *ARID1A* mutation in ccRCC was 6% in The Cancer Genome Atlas (TCGA) cohort [6], and 8% in the Renal Cell Cancer-European Union (RECA-EU) cohort [13]. Other mechanisms of ARID1A loss in RCC include deletion at 1p36 (14% [14], copy-number loss (16% [15]), and reduced mRNA expression (30% [16]) with evidence to suggest that detection of protein levels using immunohistochemistry (IHC) is a reliable surrogate marker for *ARID1A* somatic mutations [17, 18], as well as epigenetic silencing [19].

Histological assessment of tumor infiltrating mononuclear immune cells (TILs) has been shown to predict ICI response in some tumor types, with better response associated with a higher number of immune infiltrating effector T cells [20, 21]. TILs can be classified according to the spatial immune infiltration patterns as inflamed [high intratumoral immune infiltration = immune hot], non-inflamed [tumors devoid of immune cells = immune cold], and immune-excluded [lymphocytes in the peritumoral stroma only]; the latter being a rare subtype in RCC [22].

In this study, we explored the expression of ARID1A protein using IHC in a cohort of patients with advanced ccRCC who had undergone CRN and correlated our findings with clinicopathological parameters and outcome. To compare our findings to the ccRCC data set from TCGA (TCGA-KIRC) and examined the correlation between ARID1A expression and TILs as a biomarker of interest that has gained importance in translational cancer research for predicting clinical outcomes and guiding treatment.

## Materials and methods

### Patients and samples

Twenty nine patients who had undergone CRN without prior systemic treatment at the Royal Free Hospital between 2012 and 2022 were enrolled in this study. Clinicopathologic information, including age, sex, tumor size, number and location of metastasis, and follow-up data, were collected. Pathology reports and glass slides were also reviewed.

### Immunohistochemistry assay (IHC)

To confirm specificity, we optimized of the anti-ARID1A antibody (clone CL3595, Abcam, Cambridge, UK), using positive and negative control ccRCC cases with known *ARID1A*-mutation status by NGS. The anti-ARID1A mouse monoclonal antibody was then applied (dilution at 1:75) to 3–4 μm paraffin-embedded tissue sections. Slides were immunostained based on the Leica BOND IHC protocol using the Bond-Max Autostainer system (Leica Microsystems (UK) Ltd) and Bond Polymer Refine Detection kit (Leica Microsystems) (detailed staining protocol in Supplementary 1). Endothelial cells and lymphocytes served as internal positive controls for ARID1A IHC, indicating that negative staining in tumor cells was not due to technical artifacts.

Sections were qualitatively and semi-quantitatively evaluated by two investigators blinded to the clinical data, and a consensus ARID1A Histochemical-score (H-score) was calculated by multiplying the percentage of positively stained nuclei by the average intensity of staining, scored relative to ARID1A staining intensity in infiltrating lymphocytes and endothelial cells. Categories were 0 (no staining), 1 (weaker than lymphocytes and endothelial cells), 2 (moderate; equivalent to lymphocytes and endothelial cells), and 3 (stronger than lymphocytes and endothelial cells). Visual ARID1A H-scores were determined by visually averaging the intensity across the whole slide and multiplying that by the percentage of positive tumor cells. Cases which had an H-score below the level of the normal kidney median were considered ARID1A-Low, and those above were considered ARID1A-High.

### Assessment and scoring of ARID1A using QuPath digital image analysis

**Fig. 1:**
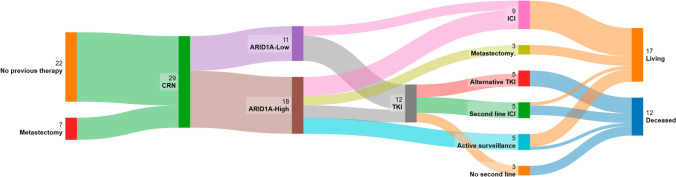
The clinical course of 29 CRN patients summarized in a river plot. In total, 22 patients proceeded straight to CRN without any pre-treatment, 7 patients had metastatic site disease excised/ablated before CRN (oligo-metastatic). 11 Patients were ARID1A-Low, 9 of them received TKI and the remaining 2 received ICI. 18 patients were ARID1A-High, 10 of them progressed and received either TKI (4 patients), ICI (3 patients) or metastasectomy (surgical, cryotherapy, or radiotherapy) (3 patients). The remaining 8 patients with stable disease either went to active surveillance (5 patients) or ICI (5 patients). Seven patients who had first-line TKI proceeded to second-line therapy in the form of an alternative TKI (4 patients) or ICI (3 patients), and 5 did not receive any second-line therapy. At the end of the study, 11 patients had died (6 of them ARID1A-Low and 5 ARID1A-High) and 18 were alive

To provide more objective assessment of ARID1A IHC, images from 3 to 5 regions of interest (ROIs) from each tumor (and normal kidney if present), were captured using an Olympus UC30 camera (Olympus, UK) at 200× magnification and analyzed using QuPath open-source software for digital pathology image analysis (version 0.5.1) [23]. Images were annotated to mark tumor areas and exclude necrotic areas, stromal areas, and tissue artifacts. Detection parameters were customized and then kept fixed for all samples (detailed in Supplementary 1, Fig. 1). Automated analysis was done using positive cell detection tool to calculate the nucleus diaminobenzidine (DAB) optical density mean of positive cells with thresholds of 1+, 2+, and 3+ to reflect varying intensities of ARID1A positive cells in the QuPath platform. The H-score (range 0–300) is given in Qupath, by calculating the ratio of the weighted sum of the number of positive cells to the total number of detected cells, thereby capturing both the intensity and the proportion of ARID1A expression from the IHC image. Automated and visual H-scores were documented and compared.

### TGCA-KIRC ARID1A gene expression and TILs analysis

For the TCGA-KIRC cohort, we obtained clinical data, processed and quality controlled files of normalized bulk RNA and genomic sequencing data for 536 cases from the Genomic Data Commons (GDC) PanCanAtlas [24], and downloaded whole-slide hematoxylin and eosin (H&E) stained diagnostic images from the Cancer Digital Slide Archive [25].

### Assessment of TILs on diagnostic H&E sections

To evaluate the tumor immune microenvironment in relation to ARID1A, TILs were assessed in one representative H&E section containing the highest-grade area in our CRN cohort, and in the diagnostic whole digital slide image (WSI) of the TCGA-KIRC cohort, blinded to ARID1A levels and clinical outcomes. TILs were visually estimated and categorized as: COLD (non-inflamed) when TILs were ≤ 10% of the tumor area; and inflamed or HOT when intratumoral TILs occupy ≥ 10% of the tumor area. The peritumoral area was not formally analyzed, because it was not represented in most of the WSI. The 10% cut-off point was defined before statistical analysis, and was selected following recommendations from breast cancer studies that linked TILs > 10% to survival [26]. TILs assessment was avoided near areas of necrosis or hemorrhage, which normally elicit a reactive inflammatory reaction regardless of tumor.

### Validation of visual TILs assessment in the TCGA-KIRC cohort

To validate the visual TILs assessment on diagnostic WSI of the TCGA-KIRC cohort, we correlated the histological classification into HOT and COLD tumors with ARID1A mRNA level, and with the Immune Score (IS) generated by the Estimation of STromal and Immune cells in MAlignant Tumors using Expression data (ESTIMATE) algorithm (https://bioinformatics.mdanderson.org/estimate/, accessed September 2024) [27]. Furthermore, TCGA-KIRC immune infiltration status generated by TIMER [28], CIBERSORT [29], CIBERSORT-ABS [30], quanTIseq (43), MCPcounter [31], xCELL [32], and EPIC [33], were download from the TIMER website (http://timer.comp-genomics.org accessed September 2024). These platforms utilize computational methods that quantify immune cells from expression data of cell mixtures using marker genes, or leveraging on deconvolution algorithms and immune cell expression signatures [34]. The immune fractions/scores from each algorithm for up to 22 TILs were correlated with ARID1A mRNA expression levels. Where the correlation was statistically significant (*p* < 0.05), the immune cell infiltration fractions were compared in ARID1A mRNA^Low^ and ARID1A mRNA.^High^ cases Additionally, we analyzed *ARID1A* in a cohort of 33 patients with advanced ccRCC, who received ICI therapy, and where both the mRNA sequencing and outcome data were publicly available [7].

### Statistical analysis

Summary statistics were used to describe the clinical and demographic characteristics of our CRN cohort, tabulated overall and by group. Frequency and percentages were used for categorical variables, and the mean (± standard deviation) or median (and interquartile range) were used for continuous variables. Differences between variables were assessed using the student *T* test (continuous), and Fisher’s exact test, or Pearson’s Chi-square test (categorical). The Kaplan–Meier curve and log-rank test were applied to assess the survival rates and to perform the univariate analysis of overall or progression-free survival (PFS). PFS was defined as the interval between the date of surgery until the date of development of new distant metastasis. Overall survival was defined as the time from diagnosis to death by censoring patients alive at their last follow-up date. Multivariate analyses were performed by Cox proportional hazards regression, and hazard ratio and 95% confidence intervals were reported. Immune cell fractions were compared in 2 groups using the Wilcoxon rank-sum non-parametric test. Spearman and Pearson correlations were used. All statistical analyses were performed using GraphPad Prism version 10.0.0 for Windows (GraphPad Software, Boston, Massachusetts USA), and a two-tailed *p* < 0.05 was regarded as statistically significant.

## Results

### Clinicopathologic characteristics of ccRCC patients

Twenty nine patients with treatment-naïve ccRCC were analyzed in this study (Table 1), including 22 men and 7 women. The mean age was 62.6 years (range 46–80 years), and the average tumor size was 85.4 cm (range 26.0–200.0). Lymph-node metastasis was identified in 3 cases (10%), and at least one site of distant metastasis was present in all cases. Of the 29 patients included in the study, 6 were categorized as pT2 (20.7%), and the remaining 23 had pT3 disease (79.3%). The ISUP nuclear grade was either grade 1 or 2 (58.6%) in 17 patients, while 6 cases were grade 3 or grade 4 (20.7% each).

**Table 1:** Association between nuclear expression of ARID1A and clinicopathological features, tumor infiltrating lymphocytes (TILs), and post-CRN management

	All patients	ARID1A-low	ARID1A-high	Univariate analysis *p* value	Multivariate analysis *p* value
Number of cases	29	11 (32%)	18 (68%)		
Gender, *n* (%)				0.676	0.7417
Male	22	9 (81.8%)	13 (72.2%)		
Female	7	2 (8.2%)	5 (27.8%)		
Age (years)	62.6	63.2 ± 9.82	62.2 ± 8.06	0.71**†**	0.7734
Number deceased	12	7 (63.6%)	5 (27.8%)	0.149	
Number of metastatic sites at final follow-up				0.449	0.8257
1 site	14	4 (28.5%)	10 (71.5%)		
2 or more	15	7 (46.7%)	8 (53.3%)		
Tumor size (cm)	85.4	105.3 ± 54.2	73.33 ± 23.8	**0**.027*	0.4492
ISUP grade				0.135	0.3142
1	6	0 (0%)	6 (33.3%)		
2	11	5 (45.5%)	6 (33.3%)		
3	6	4 (36.3%)	2 (11.1%)		
4	6	2 (18.2%)	4 (2.9%)		
3–4, *n* (%) vs 1–2		6 (54.5%)	6 (22.2%)	0.438	
Fat invasion, *n* (%)	17	8 (72.7%)	9 (50%)	0.273	
Vascular invasion, *n* (%)	19	10 (100%)	9 (50)%	**0**.024*	0.0981
Necrosis	14	7 (62%)	7 (38%)	0.196	
Stage^‡^				**0**.0142*	
1b	3	0 (0%)	3 (16.7%)		
2a	3	0 (0%)	3 (16.7%)		
3a	2	9 (82%)	12 (66.7%)		
3b	2	2 (18%)	0 (0%)		
4	0	0	0		
TILs assessment				**0**.0028*	**0**.0199*
Hot	22	5 (22.7%)	17 (77.3%)		
Cold/excluded	7	6 (85.7%)	1 (14.3%)		
Management post-CRN					
Active surveillance	5	0 (0%)	5 (100%)	**0**.012*^§^	0.305
Surgery/ablation of oligo-metastasis	3	0 (0%)	3 (100%)		
TKI first line	12	9 (75%)	3 (25%)		
TKI first line then ICI	5	3 (60%)	2 (40%)		
TKI first line then TKI + ICI	4	3 (75%)	1 (25%)		
ICI first and second line	9	3 (33.6%)	6 (66.6%)		

*p < 0.05 considered as significant and shown in bold

†Using unpaired *t* test for to compare the mean of the 2 groups. The remaining criteria were compared using the Fisher’s exact test

‡Chi-square test for trend used

§Comparison between 2 patient groups: those who did not receive targeted therapy (active surveillance + surgery/ablation = total 8) and patients who received targeted therapy of any type (total 21)

The mean follow-up period was 82.2 months (range 2–163 months). Prior to CRN, all patients had no prior therapy, but 7 had metastasectomy (surgical excision, radiotherapy, or cryoablation of metastatic deposits) prior to CRN. The mean follow-up period was 52 months (5–28 months).

At the end of follow-up, the number of metastases in each patient ranged from 1 to 5 sites (mean = 2 sites), with lung being the most common site of metastasis (19 patients), followed by bone (13 patients), liver (7 patients), and adrenal gland (4 patients). Time to first-line targeted therapy in 21 patients was 7 months on average (range =  < 1 month–28 months), while 5 patients remained on active surveillance and 3 had surgery/ablation of new metastatic deposits (Fig. 1). Nine of the 21 patients proceeded to second-line therapy in the form of ICI and/or TKI. AT the conclusion of the study, 11 patients were deceased and 18 were alive.

### ARID1A immunoreactivity

**Fig. 2:**
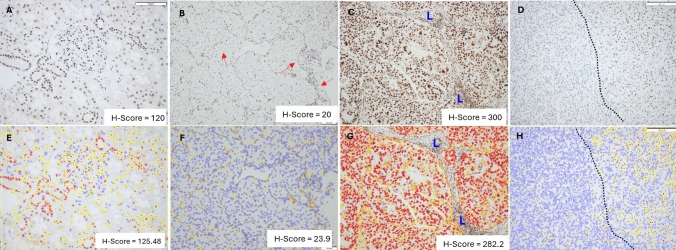
ARID1A H-Scores in ccRCC determined manually (**A**–**D**) relative to endothelial cells and infiltrating lymphocytes as reference for moderate staining. **A** Normal kidney showed weak-to-moderate intensity in the proximal tubules and strong intensity in the distal tubules. **B** ARID1A-Low case with nuclear staining in the chicken-wire network of endothelial cells (arrows) and very low expression in tumor nuclei. **C** Some tumors showed diffuse strong (3+) nuclear staining with high H-scores double those obtained in normal renal tissue. Stroma infiltrated by lymphocytes was annotated (L) and excluded from analysis. **D)** Heterogenous ARID1A IHC was zonal in 5 cases, where part of the tumor was negative (left) collides with a positive area (right). The performance of QuPath IA analysis software (**E**–**F**) was comparable to manual ARID1A H-scores, as demonstrated here in the same tumor areas. Blue nuclei = negative, yellow nuclei (1+), orange nuclei (2+), and red nuclei (3+)

Positive ARID1A protein nuclear expression was detected in all normal kidney tissues (Fig. 2A) with a median H-score of 120 (range 100–160, mean ± SD = 121.5 ± 20.3). The median H-score in normal kidney was used to dichotomize ccRCC cases into ARID1A-low and ARID1A-high. Based on this, there were 11 ARID1A-low tumors (Fig. 2B), and 18 ARID1A-high tumors (Fig. 2C). No tumor was entirely negative for ARID1A. Interestingly, in 5 out of 29 cases, there was Heterogenous expression of ARID1A, with zonal loss of ARID1A in one area colliding with a positive area (Fig. 2D). In most cases, however, there was a mixture of negative and positive nuclei within the same area.

## Concordance between manual quantification and QuPath digital image analysis

There was strong and direct correlation between the visually assigned H-scores and the QuPath IA algorithm H-score (*r*(27) = . 0.9336, *p* < 0.0001) (Fig. 2E–H, Supplementary 2, Table 1, and Supplementary 1, Fig. 2). Importantly, all the cases remained within their respective category as ARID1A-low or ARID1A-high whether the H-score was assessed manually or by QuPath. Compared to visual ARID1A H-scores, the mean QuPath H-scores for the entire cohort were higher (mean difference = 4.4; SD ± 29.5) and this was more pronounced in ARID1A-high cases (mean difference = 6.57; SD ± 34.22), while in the ARID1A-low group, the average QuPath H-scores were marginally less than manual H-scores (mean difference = − 0.675; SD ± 18.61).

### Correlations between ARID1A H-scores and clinicopathological parameters

Clinicopathologic features according to the expression patterns of ARID1A are summarized in Table 1. On univariate analysis, decreased expression of ARID1A was significantly associated with larger tumor size (*p* = 0.027), the presence of LVI (*p* = 0.024), and higher tumor stage (*p* = 0.0142). Patients who had high expression of ARID1A were significantly less likely to have subsequent oncologic/targeted therapy, compared to ARID1A-Low patients (*p* = 0.012). No other parameter was statistically associated with ARID1A expression on univariate or multivariate analyses, including tumor grade, number of metastatic sites, or the presence of necrosis.

### TILs in our CRN cohort

**Fig. 3:**
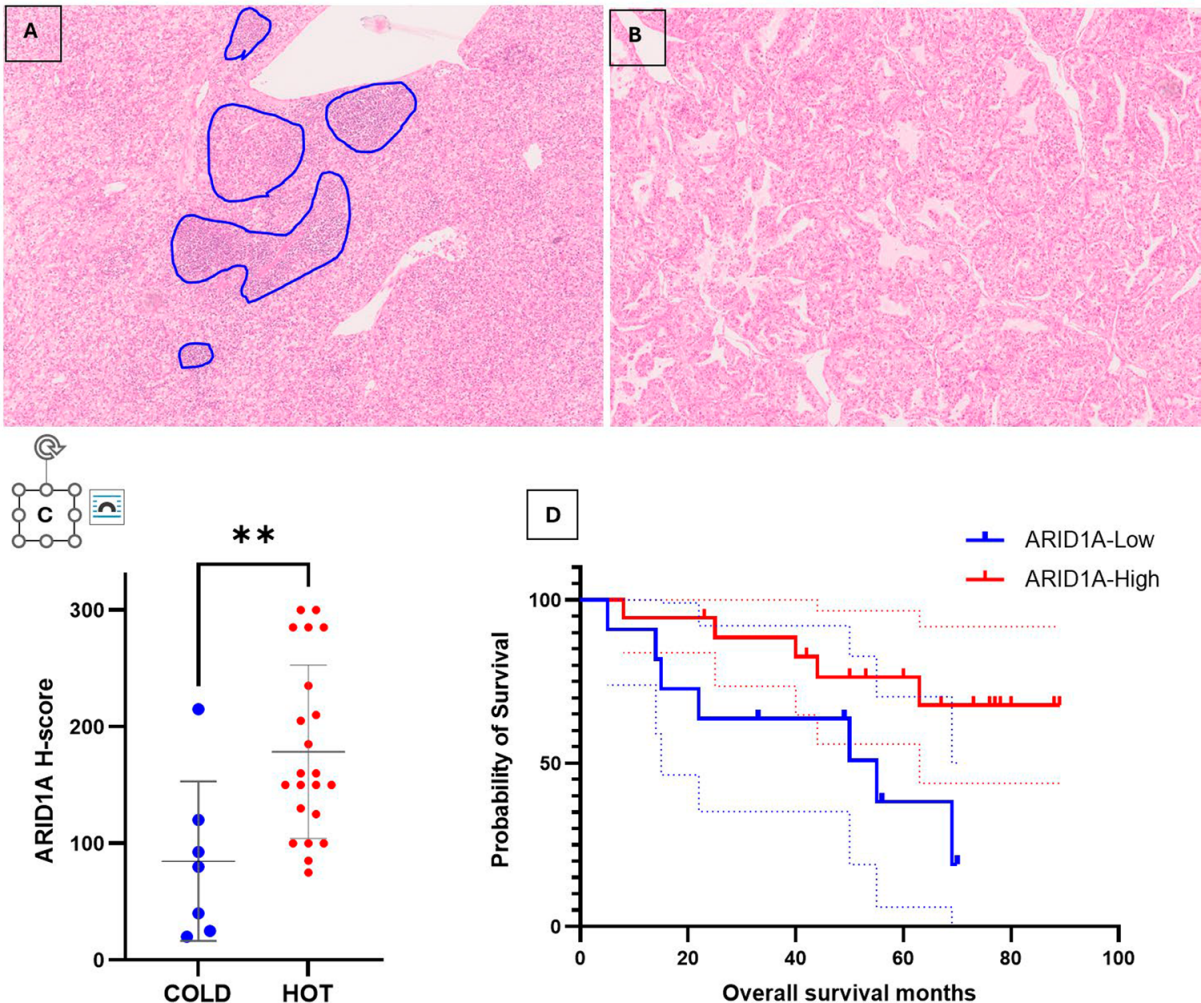
ARID1A protein expression and TILs and overall survival in the CRN cohort. **A** Immune HOT ccRCC are composed of sheets of clear cells, infiltrated by lymphocytes, macrophages, and other inflammatory cells. These may be scattered within the tumor or present in aggregates (circled), in 10% or more of the tumor area, while **B** immune COLD ccRCC contain very scanty immune cells that represent 0–10% of the tumor area. The tumor periphery was not assessed. **C** The mean ARID1A H-score in HOT tumors was significantly higher than in immune COLD ccRCCs (*p* = 0.0049). **D** ARID1A-High tumors showed significantly longer overall survival compared to ARID1A-Low tumors (log rank = 4.442, *p* = 0.0351), with > 50% of ARID1A-Low patients deceased at 5 years (95% CI shown)

TILS were assessed in the tumor area and most tumors were immune-hot (22/29 cases). The majority of these immune-hot tumors were ARID1A-High (17/22 cases). The remaining cases were immune-cold (7 cases) and these were almost all ARID1A-Low. The association between high ARID1A protein expression and immune-hot tumors was significant in both univariate and multivariate analyses (*p* = 0.0028 and *p* = 0.0199, respectively) (Table 1; Fig. 3A, B). Immune-cold tumors had a mean ARID1A H-score of 84.64 [± SE 25.85], while immune-hot tumors had a mean score of 178.4 [± SE 15.82]. This difference was statistically significant on non-parametric Wilcoxon rank-sum test (*p* = 0.0049) (Fig. 3C).

### Survival analysis in CRN

**Fig. 4:**
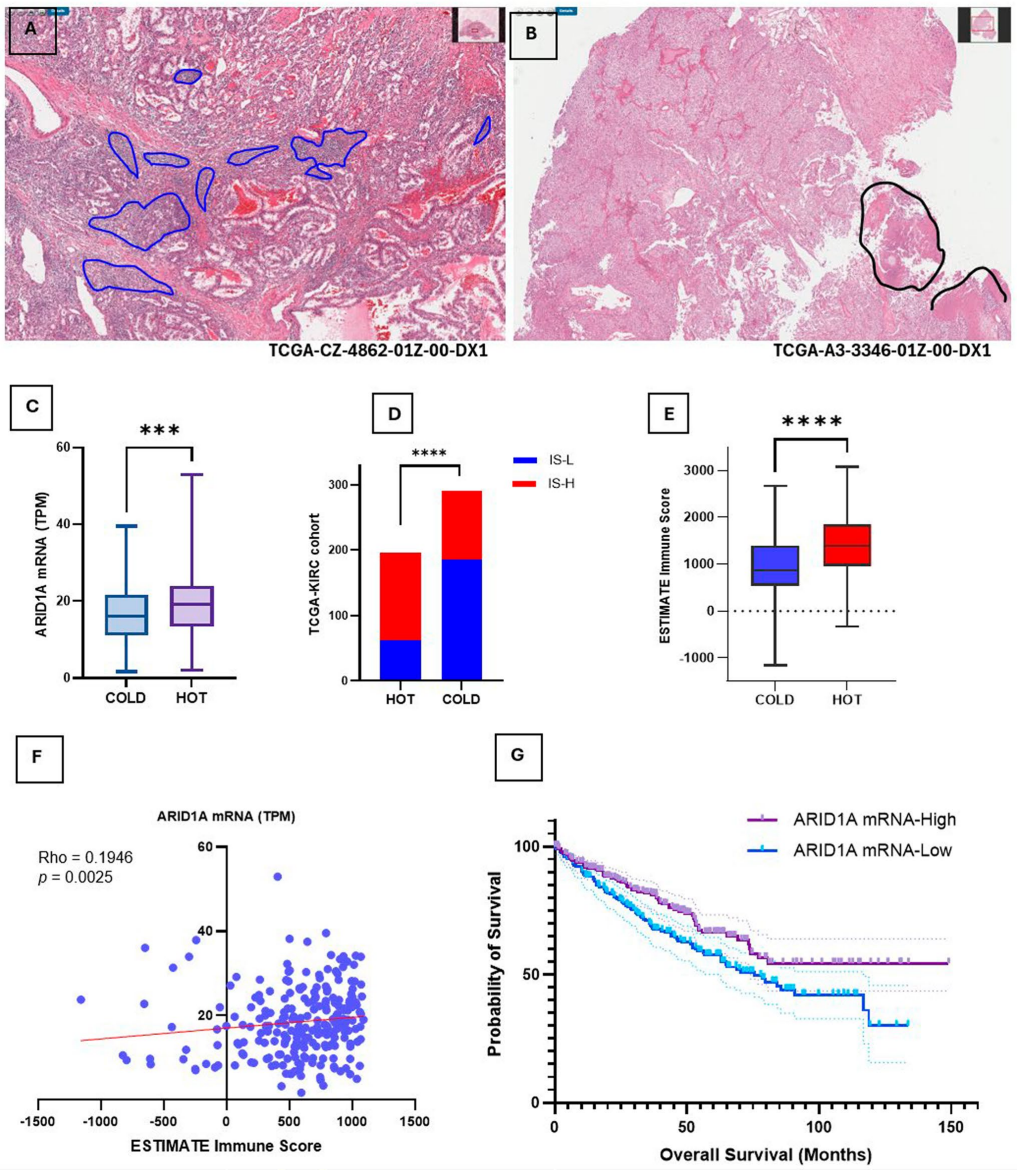
ARID1A mRNA expression in relation to TILs and overall survival in the TCGA-KIRC cohort. **A** Immune HOT ccRCC contained TILs (circled in blue) in 10% or more of the tumor area, while **B** immune COLD ccRCC contains 0–10% TILs. TILs assessment was avoided in tumor adjacent to necrotic areas (circled in black). **C** The mean ARID1A mRNA expression (TPM) in HOT tumors was significantly higher than in immune COLD ccRCCs (*p* = 0.0005). **D** Immune HOT tumors constituted 68.4% of immune score high (IS-H) cases and this was significant. **E** The mean IS was higher in immune HOT tumors compared to immune COLD, and this was highly significant (*p* < 0.0001). **F** ARID1A mRNA expression and IS showed significant positive correlation (Rho = 0.1946, *p* = 0.0025). **G** Patients who have higher ARID1A mRNA expression had longer overall survival compared to tumors with low ARID1A mRNA (log rank = 4.442, *p* = 0.0351), with > 50% of ARID1A mRNA^Low^ patients deceased at 5 years

ARID1A IHC expression had no significant influence on Progression-Free Survival (PFS) with a hazard ratio (HR) of 2.04 (95% confidence interval (CI) 0.764–5.466) in ARID1A-Low cases (*p* = 0.1067) (Supplementary 1, Fig. 3). However, ARID1A-Low patients had a significantly shorter overall survival compared to ARID1A-High cases with an HR of 3.207 (95% CI 0.9371–10.97) [*p* = 0.0338]; Fig. 3D). However, when controlling for other clinicopathological and prognostic variables including age, sex, tumor size, ISUP grade, necrosis, vascular invasion, overall stage, number of metastasis, and TILs, ARID1A protein expression did not retain its clinical significance as a marker of better overall survival on multivariate analysis (HR 1.007, CI 0.09952–7.987, *p* = 0.3610). Kaplan–Meier analysis showed that patients with immune-cold tumors had worse survival compared to those with hot tumors regardless of the ARID1A status, but this was not statistically significant [*p* = 0.1472 (Supplementary 1, Fig. 4)].

### ARID1A in TCGA-KIRC cohort

Next, we examined the prognostic significance of ARID1A mRNA expression in the TCGA-KIRC ccRCC patients. After review of the diagnostic WSI, 48 cases were excluded from analysis: 15 cases lacked ccRCC morphology and its cytogenetic hallmarks (3p deletion and von Hippel-Lindau (VHL) mutation); 28 cases had no diagnostic H&E-stained slides, 5 cases had insufficient/no identifiable tumor, or showed significant artifact.

Consequently, H&E-stained diagnostic digital WSI of 488 TCGA-KIRC cases were classified into HOT and COLD tumors using the same criteria outlined above (Fig. 4A, B; Supplementary 2, Table 2). Overall, the proportion of immune-cold tumors was higher in the TCGA-KIRC dataset compared to the CRN cases (59.3% vs. 34.5% in our CRN cohort) and the proportion of immune-hot tumors lower (40.7% vs. 65.5%). The mean ARID1A mRNA expression in immune-cold tumors was 16.83 [± SE 0.447], and 19.34 [± SE 0.57] in immune-hot tumors. This difference was statistically significant (*p* = 0.0005) (Fig. 4C).

Previous studies indicated that *PBRM1* mutations are associated with an immune-cold topography in ccRCC [35, 36], so we excluded 119 cases that harbored *PBRM1* mutations (27.8%) from TILs assessment. In *PBRM1*-proficient tumors, the mean ARID1A mRNA expression in immune-cold tumors was 16.83 [± SE 0.447], while in immune-hot tumors, the mean ARID1A mRNA was 19.34 [± SE 0.5]. The difference between ARID1A mRNA levels in HOT and COLD tumors remained statistically significant (*p* < 0.0001), indicating that ARID1A is linked to TILs in ccRCC independent of *PBRM1*.

Next, we corroborated the histologically assigned TILs classification in TCGA-KIRC tumors with the ESTIMATE Immune Score (IS). First, we divided the 488 ccRCC cases into 2 groups at the median, immune score high (IS-H) and immune score low (IS-L). Out of 241 IS-H cases, 135 were classified as immune-hot histologically (68.4%), and in 247 IS-L cases, 185 (63.6%) of cases were histologically immune-cold COLD. This was statistically significant (*p* < 0.001) (Fig. 4D). The median ESTIMATE IS was significantly higher in immune-hot tumors than in immune-cold (*p* < 0.001) (Fig. 4E). Furthermore, there was a small, but significant direct correlation between ARID1A mRNA expression and ESTIMATE IS (Rho = 0.1946, *p* = 0.0025) (Fig. 4F).

*For survival analysis*, TCGA-KIRC tumors were divided into 2 groups: ARID1A mRNA^Low^ and ARID1A mRNA^High^, each containing 244 patients. There was no significant difference in PFS of ARID1A mRNA^Low^ and ARID1A mRNA^High^ patients (log rank, *p* = 0.4624), but overall survival was significantly worse in the ARID1A mRNA^Low^ compared to ARID1A mRNA^High^ patients (HR = 1.499, CI 1.103–2.038; log rank, *p* = 0.0105) (Fig. 4G). On multivariate analysis, older age at diagnosis and higher TNM stage were statistically linked to poor survival (*p* < 0.0001), but not ARID1A mRNA expression levels.

### ARID1A in an advanced RCC ICI-therapy cohort (Miao-2018) [7]

To validate our results in the TCGA-KIRC (discovery) cohort, we investigated the role of ARID1A mRNA expression in a cohort of advanced ccRCC patients, which resemble our CRN patients both clinically and biologically and received ICI therapy. Through their transcriptomic data, we correlated ARID1A mRNA levels with survival, infiltrating immune cell composition and response to ICI therapy.

**Fig. 5:**
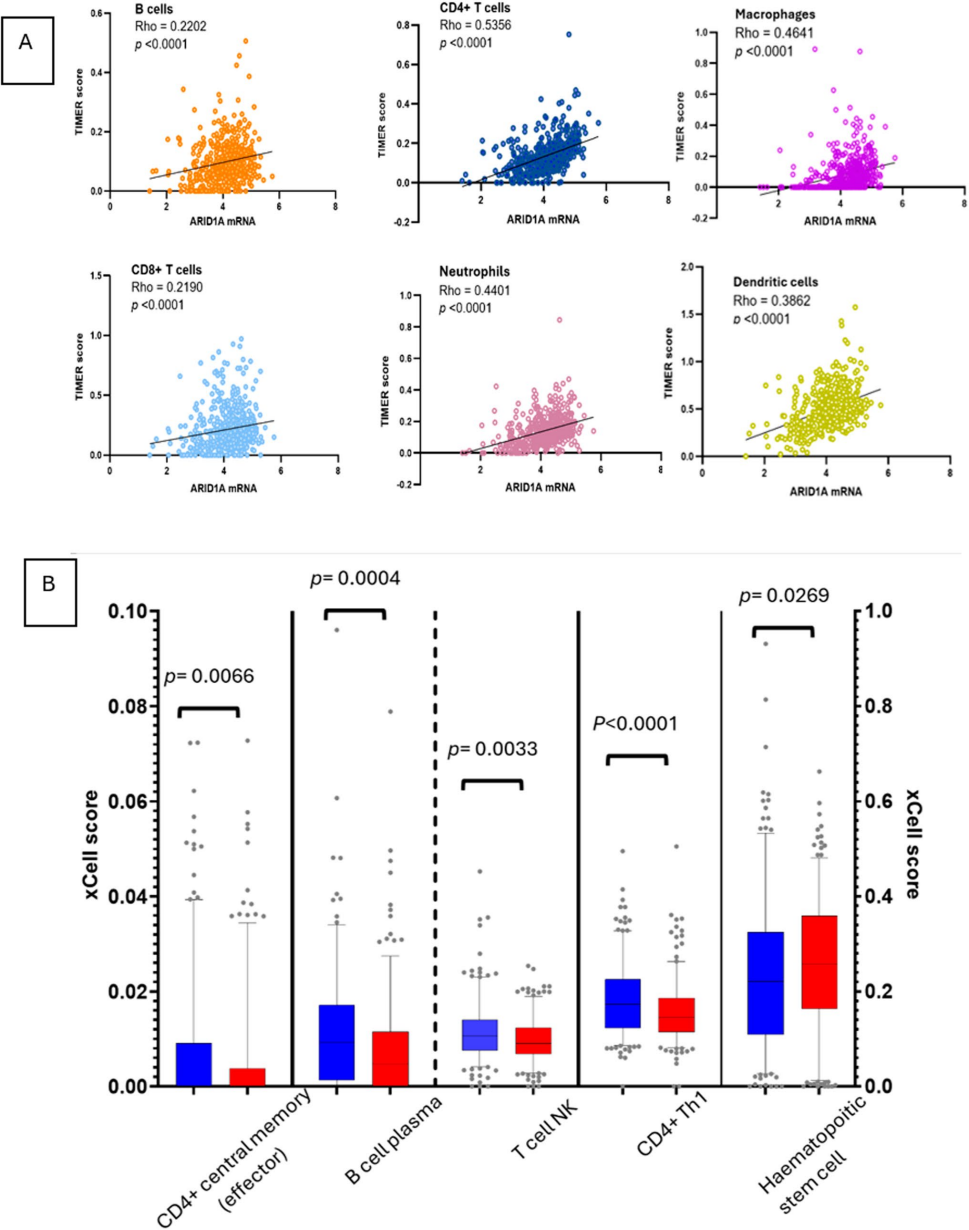
TIMER immune deconvolution analysis in TCGA-KIRC cohort provides an overview of infiltrating immune cells. **A** Positive correlation between all immune cell types and ARID1A mRNA levels. **B** When the cohort was dichotomized at the median ARID1A expression, all six immune infiltrating cell types were significantly higher in ARID1A mRNA^High^ tumors compared to the mRNA^Low^ group

**Fig. 6:**
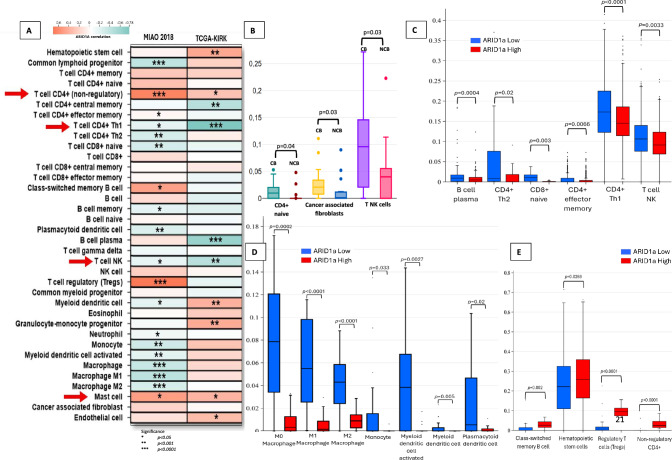
Analysis of immune infiltrates in relation to *ARID1A* mRNA levels using xCELL. **A** Correlation of ARID1a levels with immune cell populations in the Miao-2018 and TGCA-KIRK datasets demonstrated, with statistical significance highlighted with asterisks. **B** Boxplots showing significantly elevated levels of CD4⁺ T cells, cancer-associated fibroblasts, and T-cell natural killer cells in patients who derived clinical benefit from ICI therapy. **C**–**E** Immune cell enrichment patterns stratified by *ARID1A* mRNA expression in the TCGA-KIRC and Miao-2018 cohorts reveals a paradoxical increase of subsets of CD4+ lymphocytes, macrophages, and antigen-presenting cells in ARID1A mRNA^Low^. Tregs were, however, increased in ARID1A mRNA^High^ tumors

ARID1A^Low^ patients had worse progression-free and overall survival than ARID1A^High^ patients, but this was not statistically significant (Supplementary 1, Fig. 5). ARID1A mRNA levels were compared in patients who had clinical benefit (CB: defined as partial or complete response or stable disease) and those with progressive disease (NCB), but this was not statistically significant (*p* = 0.1243) (Supplementary 1, Fig. 6A). ARID1A mRNA levels showed a trend for positive correlation with levels of PD-L1 mRNA, but this was not statistically significant (*r* = 0.2183, *p* = 0.2223; Supplementary 1, Fig. 6B).

### Immune cell infiltrates and ARID1A mRNA expression levels

To estimate of immune cell infiltration beyond global histological TILs assessment and IS, 7 immune deconvolution algorithms were employed to quantify the relative proportions of immune cells in relation to ARID1A mRNA expression (Supplementary 2, Table 3). In TCGA-KIRC, most immune cell scores/fractions were positively correlated with ARID1A mRNA (Supplementary 2, Table 4 and Supplementary 1, Fig. 7A) and this was highlighted in TIMER where, in ARID1A mRNA^High^ cases, there was a significant increase in all 6 immune cell types compared to ARID1A mRNA^Low^ cases (Fig. 5).

As xCell provides more immune cell types compared to other deconvolution mechanisms, and an “absolute score” of each sample, enabling more reliable comparisons between samples, we utilized this platform to specifically interrogate correlations and inter-sample differences in immune cellular composition.

In the TCGA cohort (Fig. 6A), there was a significant positive correlation between high ARID1A mRNA levels and hematopoietic stem cells, CD4+ T cells (non-regulatory), myeloid dendritic cells, endothelial cells, granulocyte–monocyte progenitors, and mast cells, but CD4+ central memory, CD4+ Th1 cells, plasma B cells, and T NK cells were significantly reduced (also Supplementary 1, Fig. 7A).

The immune infiltration profile of patients in the Miao-2018 cohort (Fig. 6A), which represents a select group of patients with locally advanced or metastatic RCC, overlapped with TCGA-KIRC RCCs, but notably, with significant increase in Tregs and reduction in macrophages (M0, M1, and M2). ARID1A significantly and consistently correlated with four cell types in both cohorts (positive correlation with CD4+ T cells and mast cells, and negative correlation with CD4+ Th1 and T NK cells), suggesting a strong link between *ARID1A* and the recruitment/immune activity of these cells in RCC.

The transcriptomic data of the Maio-2018 cohort provided a unique opportunity to analyze the immune cell composition tumors in patients who responded to ICI (CB) and those who had progressive disease (NCB) using xCELL. This revealed a significant positive correlation of three immune cell types in patients with CB compared to those with NCB: CD4+ T cell naïve, cancer-associated fibroblasts, and T NK cells (Fig. 6B).

To evaluate whether any of these cells are linked to ARID1A, we compared the immune infiltrates of ARID1A mRNA^High^ and mRNA^Low^ in the TCGA-KIRC and Miao-2018 cohorts.

Among lymphocytic immune subsets (Fig. 6C), B-cell plasma, CD4+ Th1 and Th2, CD8 naïve, CD4+ effector memory, and T TNK cells were elevated in ARID1A mRNA^Low^ tumors. Among the myeloid linage, macrophages (M0, M1, and M2), monocytes, myeloid dendritic cells (including the activated subset), and plasmacytoid dendritic cells were all elevated in ARID1A^Low^ tumors (Fig. 6D). In ARID1A^High^, there was a significant increase in cells CD4+ cells (non-regulatory), the immunosuppressive Tregs and hematopoietic stem cells, and class-switched memory B cells (Fig. 6E).

## Discussion

In the present study, we analyzed the ARID1A protein expression in a cohort of CRN patients, matching cases with late-stage/metastatic ccRCC. We showed that in ccRCC, lower-than-normal expression ARID1A protein is associated with larger tumor size, LVI, and higher stage. These patients had shorter overall survival in univariate analysis, but not on multivariate analysis. We also demonstrated that patients with low ARID1A mRNA in the TCGA-KIRC cohort, similarly, had significantly shorter overall survival compared to patients who had high ARID1A mRNA, but on multivariate analysis, only patient age and stage were statistically linked to overall survival. This suggests that ARID1A is a marker for poor prognosis, but not an independent marker of survival, indicating that tumor characteristics and treatments patients receive may be more critical. For example, although loss of ARID1A predicts poor prognosis, it offers survival benefit to ccRCC patients who received ICI therapy compared to those who receive TKIs [10], so ARID1A’s link to survival is complex. Three previous studies have examined ARID1A protein expression in a total of 389 ccRCC patients [9, 15, 16]. Similar to our study, Lichner et al. [15], and Park et al. [16] linked loss of ARID1A expression to a larger tumor size, higher nuclear grade, and higher stage, and demonstrated that ARID1A-positive cancers exhibited a longer disease-free and overall survival in univariate and also multivariate analyses. They suggested that ARID1A expression was an independent prognostic factor for progression-free survival. Somsuan et al. [9] confirmed downregulation of ARID1A in ccRCC compared to adjacent normal tissue, and provided evidence that gene siRNA knockdown in kidney cell lines leads to increased proliferation, epithelial–mesenchymal transition, migratory activity, nuclear size, invasion capability, and chemoresistance (to docetaxel). This is consistent with the more frequently observed loss of *ARID1A* tumor suppressor function in higher grade and late-stage tumors in our study and others.

We found that higher ARID1A protein expression in our CRN cohort, and higher ARID1A mRNA expression levels in the TCGA-KIRC cohort, were both associated with increased numbers of TILs in ccRCC tumor tissue histologically. At the mRNA level, the ESTIMATE algorithm IS confirmed that ARID1A mRNA^High^ ccRCC had a higher IS than the ARID1A^Low^ group. Deconvolution platforms for cell-type enumeration profiling revealed that immune cell abundance was positively and significantly correlated with higher ARID1A mRNA expression.

Despite an overall reduction in TILs in ARID1A^Low^ cases, immune cell subtype analysis demonstrated a paradoxical and selective increase in increase in infiltrating myeloid dendritic cell, which are antigen-presenting cells that facilitate immune response, as well as plasmacytoid dendritic cells, responsible for the production of type I interferons [37]. Activated dendritic cells drive differentiation of CD4+ T cells into Th1 and Th2, both of which were also increased in ARID1^Low^ cases. In addition, ARID1A^Low^ cases had higher recruitment of macrophages (both M1 and M2). M1-polarized macrophages have anti-tumor activity. They can drive Th responses by antigen presentation, involving T-cell proliferation and IFN-γ release and can act more effectively than M2-like macrophages, especially if the latter is located within the stroma rather than intra-tumoral [38]. Indeed, gene set enrichment analysis (GSEA) of gene signatures enriched in *ARID1A mutant RCC* revealed the top enrichments as IFN Alpha and IFN Gamma Responses [39], which may explain the pattern of immune cell infiltration seen in the ARID1A^Low^ cases.

Immune responder cells (for example effector memory CD4+ T cells) were also highly enriched in *ARID1A*^Low^ patients, while immune suppressor cells such as Tregs and hematopoietic stems cells were increased in ARID1A^High^ cases. In RCC, Tregs were found to suppress tumor immune responses immunity through direct cellular contact and inhibitory cytokine secretion, promoting RCC growth, metastasis, and angiogenesis [40]. Hematopoietic stem cells are reported to have immunosuppressive and tumor-promoting properties [41] suggesting a compromised immune microenvironment despite increased TILs in these tumors. A previous study has shown that class-switched memory B cells were enriched in RCC ICI responders [42]. Its increase in ARID1A ^High^ suggests an active humoral anti-tumor response within tertiary lymphoid structures driven by B cells.

Surprisingly, our study demonstrated that ARID1A^Low^ tumors were enriched for T NK cells in both the TCGA and Miao-2018 cohort, and that this was one of the cells identified to be significantly higher in the ICI-responsive tumors compared to those with NCB in the Miao-2018 cohort. T NK cells, although innate-like, were found to migrate to tumors in response to chemokines and either kill or polarize pro-tumor M2-like macrophages into antitumor M1-like macrophages [43]. Their role in RCC in relation to ICI response and to *ARID1A* merits further analysis.

Our findings suggest an immune advantage to tumors with loss of ARID1A and may explain the better response obtained in *ARID1A*-mutant ccRCC to ICI compared to TKI in the IMmotion 151 clinical trial [registered with ClinicalTrial.gov NCT02420821 on 15.04.2015] [10].

Cytotoxic CD8+ T cells are thought to have a dominant role in responses to ICI and other forms of immunotherapy. In this study, there was positive correlation between ARID1A mRNA and CD8+ cells. In ccRCC, lung adeno- and squamous cell carcinomas, there were dramatically lower CD8^+^ T cell infiltrations in *ARID1A*-mutant tumors compared to non-mutated cases, but CD8^+^ T cells were enriched in *ARID1A*-mutatant endometrial, gastric, and colonic adenocarcinoma [44, 45]. Taken together, these seemingly conflicting results in different cancer types indicate that ARID1A-regulated antitumor immunity is cancer type and context-dependent. Interestingly, and similar to our findings in the Miao-2018 cohort, a previous study also reported that baseline CD8+ infiltration of ccRCC was not associated with response to anti-PD-1 therapy [22].

In the current study, we demonstrated that ARID1A protein expression by IHC analysis mirrors results obtained by NGS in the TCGA-KIRC dataset. Because IHC is a widely available and accessible technique, unlike NGS, our findings indicate that ARID1A IHC can be reliably applied to larger cohorts of ccRCC patients prospectively or retrospectively, to determine whether it is a potential biomarker for ICI response.

There are limitations to our research. First, our study population is small, because it only includes a select cohort of metastatic, treatment naïve ccRCC patients. These represent most ICI eligible patients but avoids potential post-treatment alterations that could influence ARID1A or TILs assessment. Of note, the outcomes of ccRCC patients in this study represent retrospective real world and may not accurately reflect the current standard of ccRCC management, where most patients are offered neoadjuvant ICI combination/systemic therapy followed by deferred CRN. Second, TILs assessment in the TCGA-database was based on the single diagnostic WSI available in the TCGA archive. Selection of the most representative TILs tumor area was therefore not possible. In addition, we were unable to specifically investigate the immune-excluded phenotype as a separate entity, since TCGA-KIRC tumor images often lacked the tumor/non-tumor interface. This limited our ability to analyze the effect of ARID1A on this type of immune dysfunction. Third, TCGA-KIRC bulk-RNA-sequencing data and immune deconvolution algorithms are susceptible to variation and background prediction, so they may not be the “gold standard” method for analyzing immune infiltrating cells in tumor microenvironment.

## Conclusion

In the era of rapidly expanding molecular analysis, establishing the clinical relevance of a potential marker requires large-scale testing of patient tissues either in a retrospective or prospective manner. This is facilitated by an open data-sharing policy of clinical trial data and online databases, the use of artificial intelligence, and multi-omics cutting-edge technology that allows bulk extraction and analysis of data [46], drug discovery [47, 48], and complementing transcriptomic data with electrophysiological analysis to understand the dynamics of the tumor microenvironment [49]. In this pilot analysis, we used IHC, a widely available and accessible technique, to demonstrate that low levels of ARID1A expression in a local cohort of advanced renal cell carcinoma patients are a marker of poor survival. These findings were consistent with data obtained by ARID1A mRNA analysis in the publicly available TCGA-KIRC cohort. Histological assessment of TILs in our cohort and in diagnostic images from TCGA-KIRC cohort confirms that low ARID1A expression, on the protein and mRNA level respectively, is associated with reduced TILs, but CD4+ helper 1 and effector memory T cells and NK cells are enriched in cases with low ARID1A, which may potentially present an advantage for patients receiving ICI. In-depth analysis of immune infiltrates employing multiplex immunofluorescence would reveal, not only the abundance, but the spatial distribution of CD4+ and CD8+ T cells subsets, which is key to understanding the immune microenvironment in ARID1A-deficient tumors. Single-cell transcriptomics and mechanistic studies using *ARID1A* cell line knockouts in mouse models may allow more refined subtyping of immune cell populations, including their functional relationships within the ccRCC tumor microenvironment in relation to ARID1A.

## Supplementary Information

Below is the link to the electronic supplementary material.

Supplementary file1 (DOCX 3164 KB)

Supplementary file2 (XLSX 1122 KB)

## Data Availability

The original contributions presented in the study are included in the article/Supplementary Material (Supplementary files 1 and 2), and further inquiries can be directed to the corresponding authors. The datasets analyzed during the current study are available in the TCGA—PanCanAtlas Publications | NCI Genomic Data Commons repository, https://gdc.cancer.gov/about-data/publications/pancanatlas.

## Abbreviations

ARID1A: AT-rich interactive domain-containing protein 1A
ASR: Age-standardized rate
ccRCC: Clear cell renal cell carcinoma
CD4: Cluster of differentiation 4
CD8: Cluster of differentiation 8
CI: Confidence interval
CRN: Cytoreductive nephrectomy
DAB: Diaminobenzidine
GDC: Genomic Data Commons
H score: Histochemical score
H&E: Hematoxylin and eosin
HR: Hazard ratio
ICI: Immune checkpoint inhibitors
IHC: Immunohistochemistry
IS: Immune Score
IS-H: Immune score high
IS-L: Immune score low
ISUP: International Society of Urological Pathology
LVI: Lymphovascular invasion
NK: Natural killer
PBRM1: Polybromo 1
PD-L1: Programmed death-ligand 1
PFS: Progression-free survival
RCC: Renal cell carcinoma
RECA-EU: Renal Cell Cancer-European Union
ROI: Regions of interest
TCGA-KIRC: The Cancer Genome Atlas-Kidney renal clear cell carcinoma
Th 1: T-helper cell 1
TILs: Tumor infiltrating mononuclear immune cells
TKI: Tyrosine kinase inhibitors
Treg: T-cell regulatory
VHL: Von Hippel-Lindau
WSI: Whole slide image
