# Supplementary material for: Loss of ARID1A expression is associated with worse survival and reduced tumor infiltrating lymphocytes in advanced clear cell renal cell carcinoma
Source: Clin Transl Oncol. 2026 Jan 3;28(6):2345–59. doi: 10.1007/s12094-025-04166-8 (PMC13186821; doi:10.1007/s12094-025-04166-8)
Supplement: Supplementary file 1 — Supplementary file1 (DOCX 3164 KB) [file 12094_2025_4166_MOESM1_ESM.docx]

Supplementary material and methods

Immunnohistochemistry staining protocol

ARID1A is a nuclear antigen, defined by its reactivity with ARID1A antibody (clone CL3595, Abcam, Cambridge, UK). ARID1A antibody was optimised using the manufacturers recommendation of antigen retrieval. Several dilutions were preformed to compare strongest ARID1A with the weakest non-specific background staining and was optimised to a 1:75 dilution (when applied on FFPE sections of a normal kidney and using HIER, pH 6 (citrate).

Formalin fixed paraffin embedded tissue blocks were sectioned at 3mm thickness using a Leica RM2235 rotary microtome and mounted on Leica Bond Plus slides, for ARID1A staining.

The slides were first placed in a 37°C oven for 20 minutes then loaded into the Leica Bond staining machine the staining order is as follows:

1. Dewaxing 5 minutes

2. Antigen retrieval for 40 minutes at pH 6

3. Peroxidase block

4. Incubated for 30 minutes in primary antibody at 1:75 dilution

5. Leica Bond Polymer Refined kit

6. Counterstain with haematoxylin

7. Blueing solution

8. Slides dehydrated and cleared alcohol and xylene respectively, then mounted

QuPath Image analysis

Full instructions for the software are available here: <https://qupath.readthedocs.io/en/stable/docs/starting/first_steps.html>

Initially, a control section was used as a training image for colour deconvolution using the ‘estimating stain vectors’ command in QuPath to optimise haematoxylin, 3,3′-diaminobenzidine (DAB), and background detections. The image was annotated using the polygon tool to limit the analysis to viable tumour cells. Stroma and immune cells were classified using the object classifier trained on all prior detections using a random trees classifier, with all feature and class measurements included. The classifier was then saved.

Briefly, the test image file is then opened and image type “Brightfield (H-DAB) image” is selected. In the analysis panel, the “cell detection” function was applied to detect ARID1A positive nuclei using the command: Analyze ‣ Cell detection ‣ Positive cell detection

The following parameters were used in all cases:

| Setup Parameters | |
| --- | --- |
| Detection image | Optimal density sum |
| Nucleus Parameters | |
| Background radius | 6 pixel |
| Median filter radius | 0 pixel |
| Sigma | 1.5 μm |
| Minimum area | 10 pixel ^2^ |
| Maximum area | 400 pixel ^2^ |
| Intensity Parameters | |
| Threshold | 0.1 |
| Maximum background intensity | 2 |
| Split by shape | Selected |
| Exclude DAB (membrane staining) | Not selected |
| Cell Parameters | |
| Cell expansion | 5 pixel |
| Include cell nucleus | Selected |
| General Parameters | |
| Smooth boundaries | Selected |
| Make measurements | Selected |
| Intensity Threshold Parameters | |
| Score compartment | Nucleus: DAB Optical Density mean |
| Threshold 1+ | 0.2 |
| Threshold 2+ | 0.4 |
| Threshold 3+ | 0.6 |
| Single Threshold | Not selected |

Pressing the “run” button, will then initiate the analysis and the results are revealed as the total number of objects detected, the total number and percentage of positive nuclei, and the number of nuclei identified in each threshold. The overall H-score is also given.


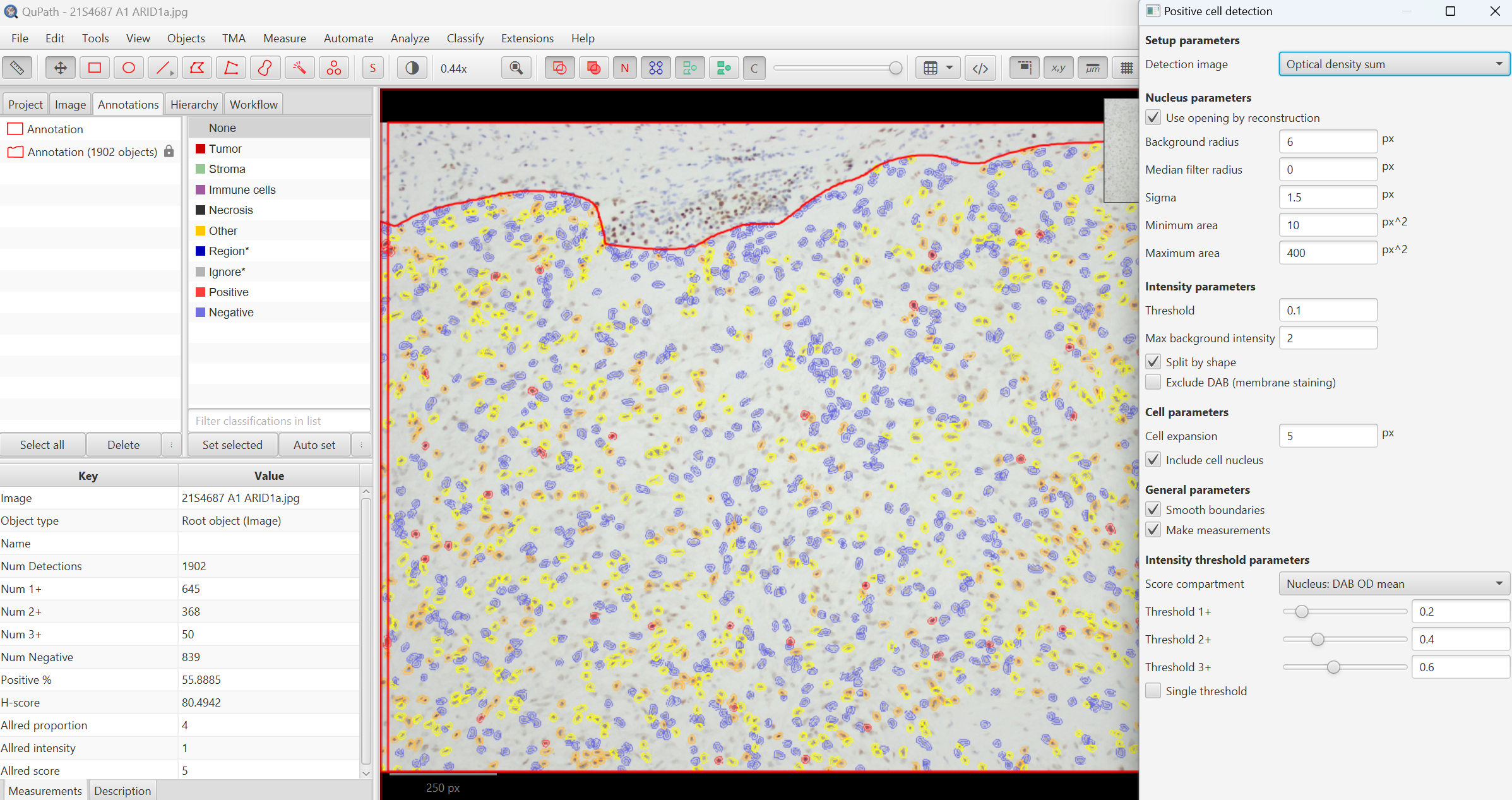


Supplementary Figure 1. Optimised positive cell detection settings for determining ARID1A expression status in ccRCC cases (29 CRN) using the QuPath image analysis software shown in the right-sided panel. Only cells within the annotated tumour area are scored, while stromal area are ignored even if they contain positive lymphocytes and endothelial cells (top). On the left, the breakdown of ARID1A staining is broken down by intensity and percentage, then an overall weighted H-score is given.


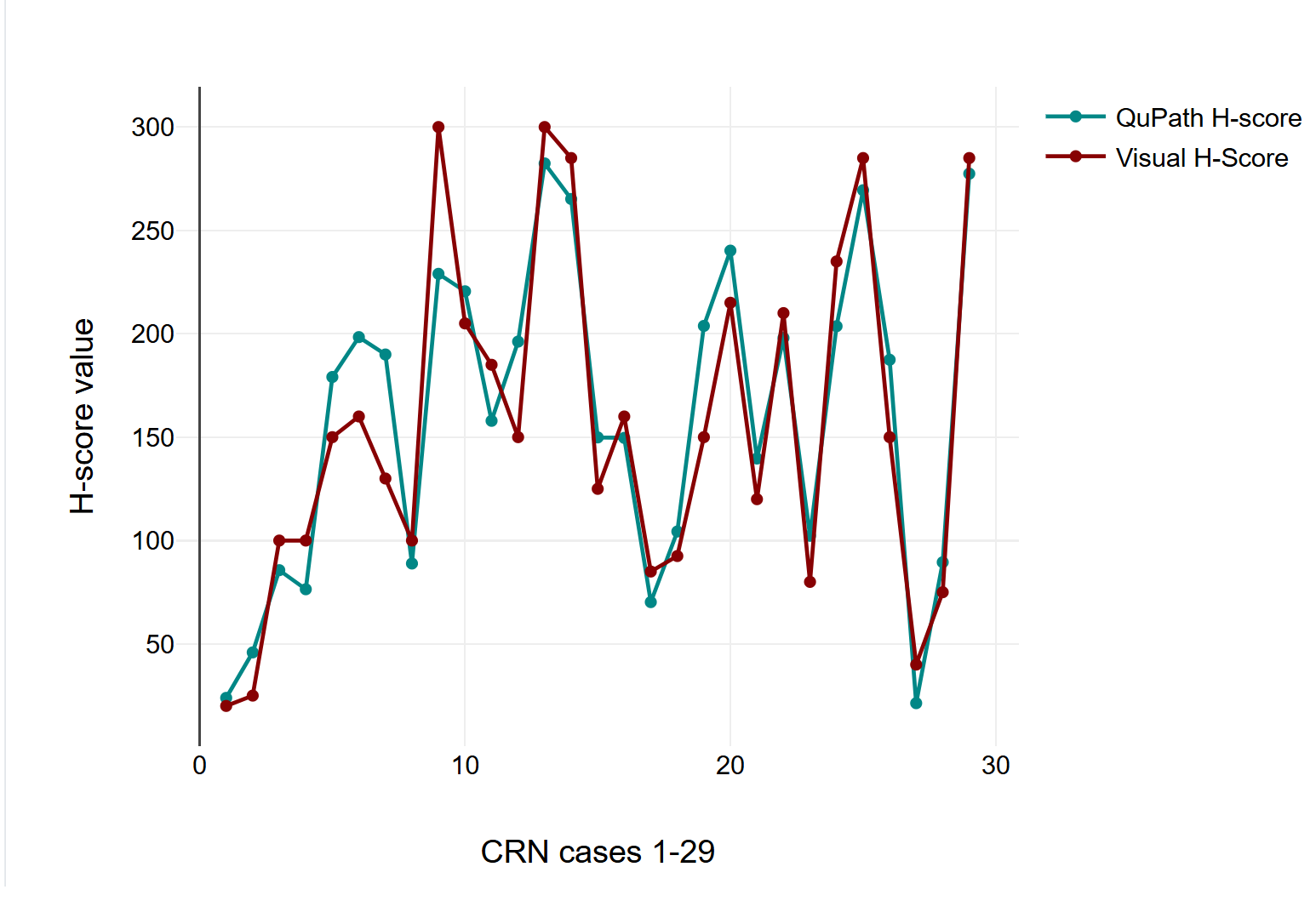

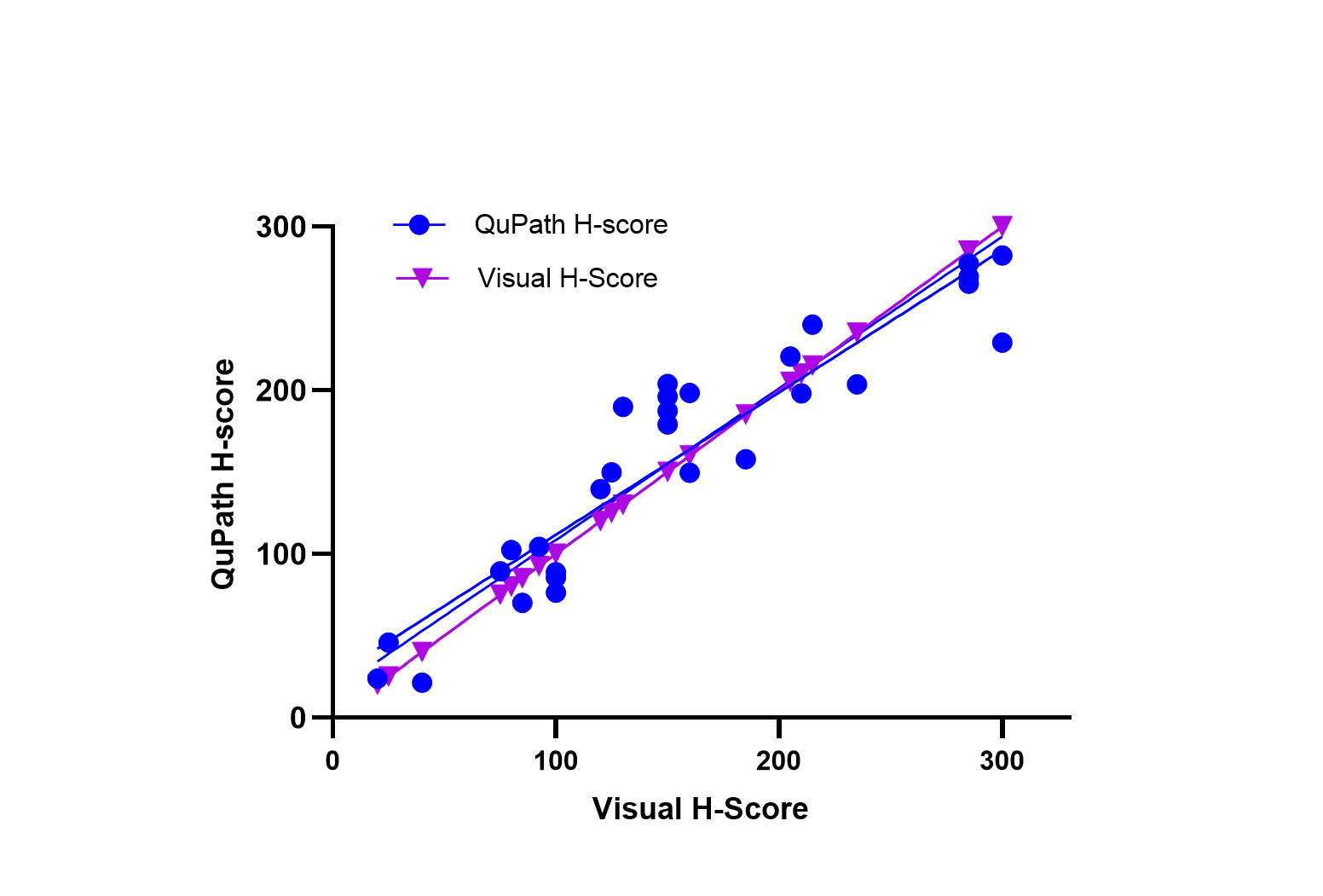


A

B

Supplementary Figure 2. Comparison between manual assessment of ARID1A IHC H-scores and the QuPath scores for CRN ccRCC cases. A. Line plot showing the Visual versus Qupath H-scores obtained from each case. B. Correlationbetween Visual H-scores and QuPath H-score. Spearman’s *r_s_* = 0.93136, *p* <0.0001.

Supplementary Figure 3. ARID1A and Progression Free Survival (PFS) in the CRN cohort. There was poor PFS in patients with low ARID1A expression compared to those with high expression but this was not statistically significant on the log rank test *p* = 0.1067

Supplementary Figure 4. TILs and overall survival in the CRN cohort. There was poor overall survival in patients with low TILs and immune cold tumours compared to those with immune HOT tumours, but this was not statistically significant on the log rank test *p* = 0.1472


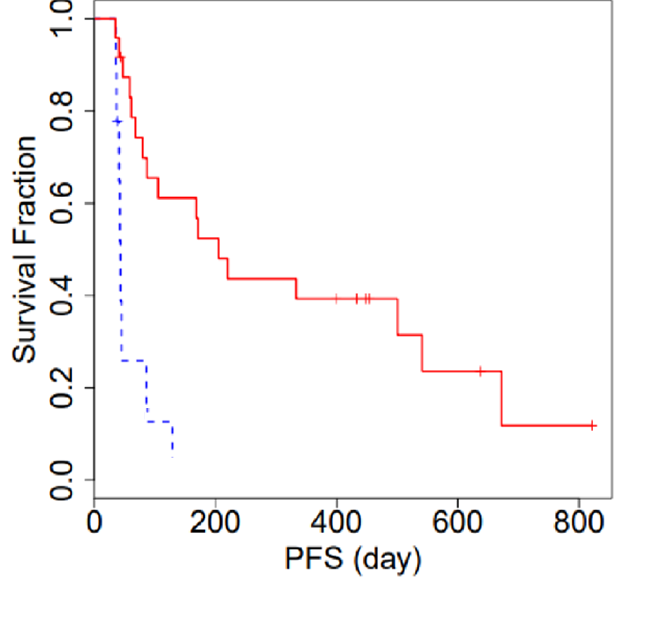


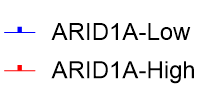


Supplementary Figure 5. Progression-Free survival in the Miao-2018 cohort. There was poor overall survival in patients with low ARID1A mRNA but this was not statistically significant on the log rank test *p* = 0.112


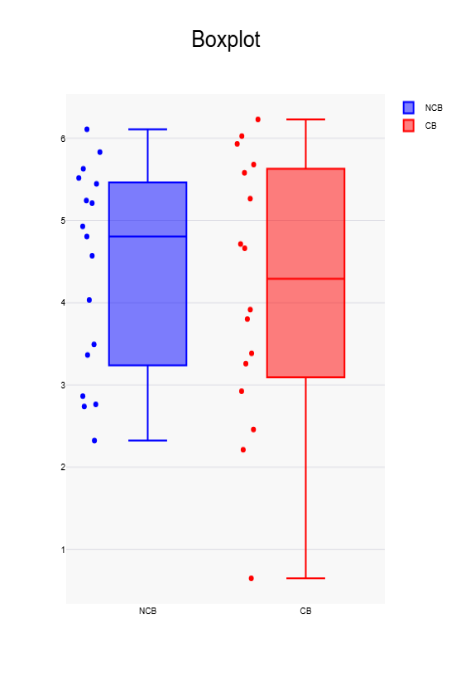


B

A


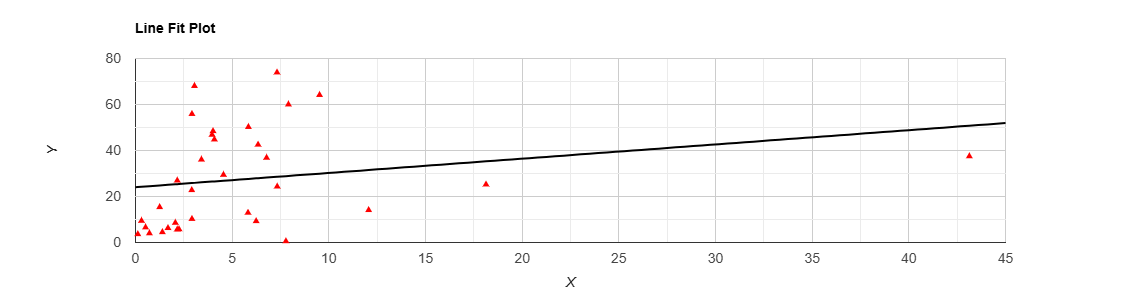


Figure 6. In the ICI Miao cohort, (A) there was no significant difference between the levels of ARID1A mRNA in patients who benifited frim ICI therapy (CB) versus those who did not (NCB) p = 0.1243. (B) there was a positive but insignificant correlation between the mRNA levels of ARID1a and PD-L1 in these patients (**r** = 0.2183, p = 0.2223).


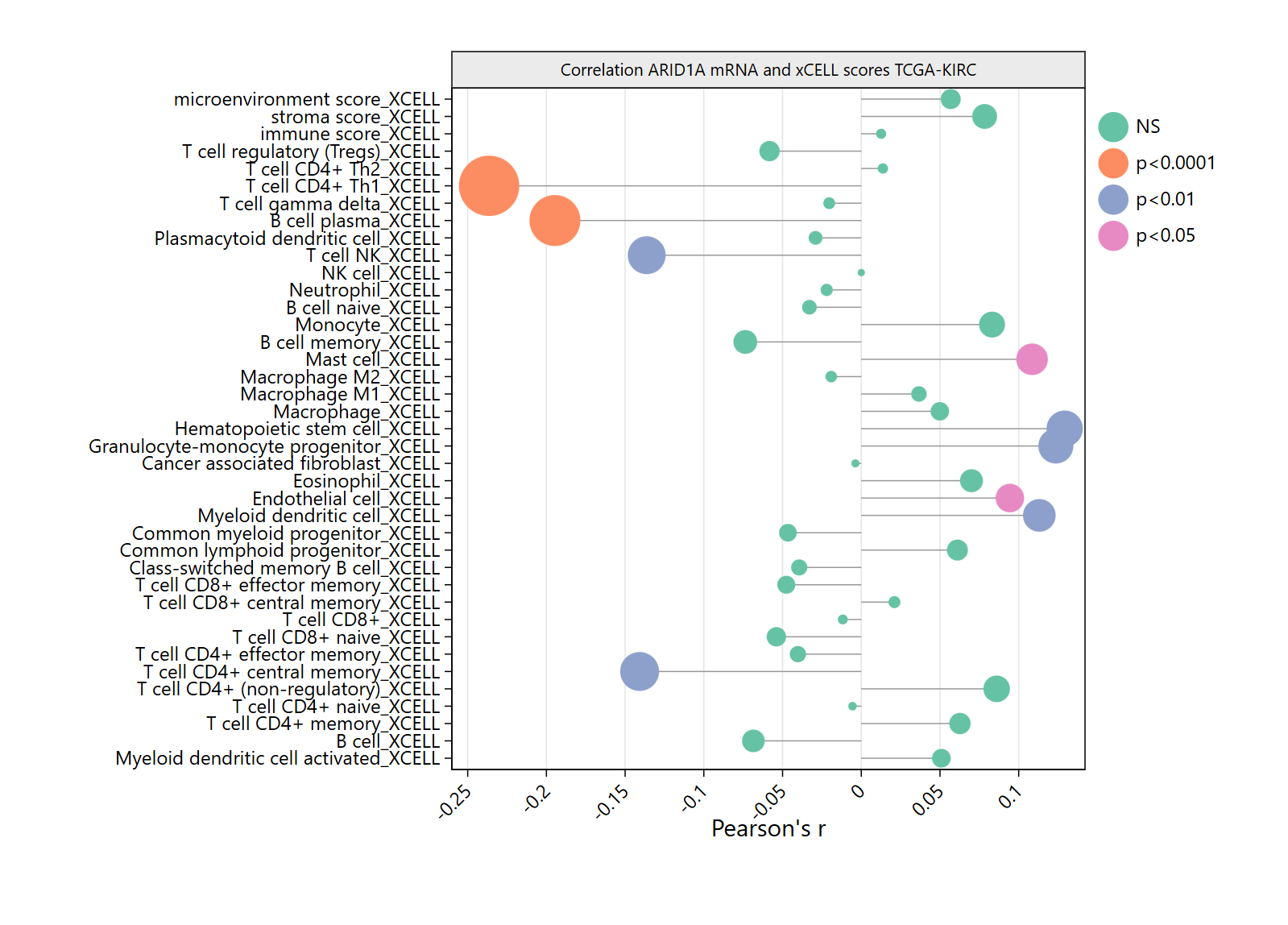


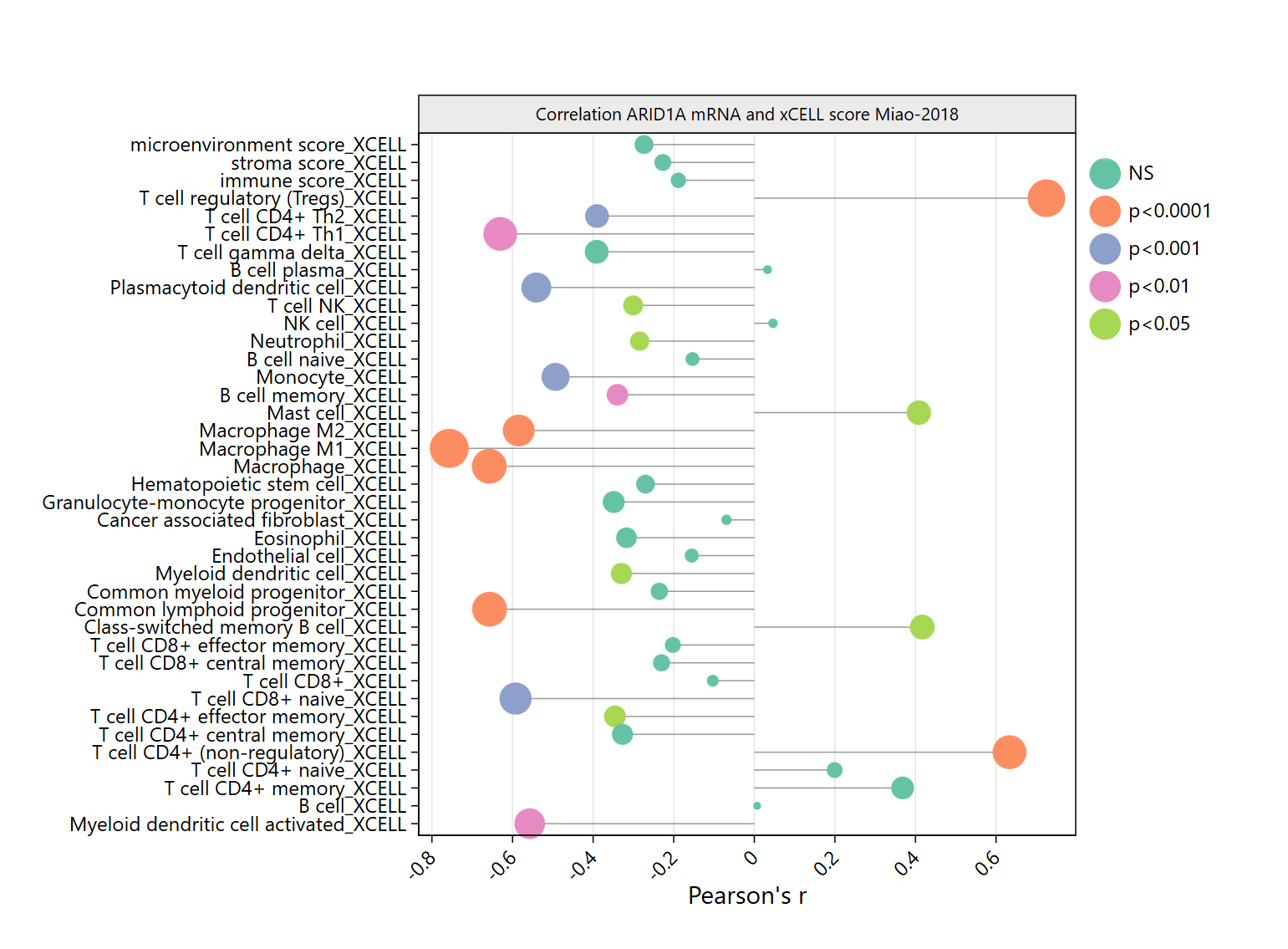


Supplementary Figure 7. ARID1A mRNA correlation analysis in the TCGA-KIRC cohort (A) and (the Miao-2018 cohort (B), reveals differences and overlaps. Notably, more immune cells were negatively correlated with ARID1A expression in the Miao-2018 cohort, likely reflecting that the Miao cohort are a select group of patients with advanced RCC compared to the TCGA-KIRC, which includes many cases of low grade and stage.
